# Optimized Extraction, Comprehensive Chemical Profiling, and Antioxidant Evaluation of Volatile Oils from *Wurfbainia villosa* (Lour.) Škorničk. & A.D.Poulsen Leaves

**DOI:** 10.3390/plants14132041

**Published:** 2025-07-03

**Authors:** Yuancong Gu, Bangyu Lv, Xingrui Nian, Xinrui Xie, Xinhe Yang

**Affiliations:** College of Food Science and Engineering, Guangdong Ocean University, Yangjiang 529500, China; guyuancong33@163.com (Y.G.);

**Keywords:** *W. villosa* leaves, cellulase, extraction, GC-MS, chemical constituents, antioxidation

## Abstract

This study employed cellulase-assisted hydrodistillation (cellulase-HD) to extract volatile oils from *Wurfbainia villosa* (Lour.) Škorničk. & A.D.Poulsen *(W. villosa)* leaves, with process optimization conducted via the response surface methodology (RSM). The optimized extraction parameters were as follows: enzyme dosage 2.2%, enzymatic hydrolysis temperature 49 °C, hydrolysis duration 73 min, and material/liquid ratio (1:10.7 mg/mL). Under these optimal conditions, the volatile oil yield reached 0.772%, representing a 31.29% increase compared to conventional hydrodistillation (HD). GC-MS analysis identified 54 and 49 volatile compounds in cellulase-HD and HD extracts, respectively, with 39 shared components. The cellulase-HD extract was predominantly composed of γ-terpinene (14.981%), limonene (13.352%), β-phellandrene (10.634%), 4-terpineol (10.145%), and α-terpineol (8.085%). In contrast, the HD extract showed higher contents of β-phellandrene (41.881%), followed by β-myrcene (8.656%) and limonene (8.444%). Notably, cellulase pretreatment significantly increased the yield of oxygenated compounds. Orthogonal partial least squares discriminant analysis (OPLS-DA) revealed substantial compositional differences between the two extraction methods, with key differential components including fenchol, borneol, and γ-elemene. Antioxidant activity assessment demonstrated superior free radical scavenging capacity in cellulase-HD extracts. Structure–activity relationship analysis identified seven compounds with DPPH radical scavenging rates >50%, particularly, epi-bicyclosesquiphellandrene (71.51%) and γ-elemene (78.91%). Furthermore, thirteen components, including isopinocamphone (66.58%) and α-terpineol (66.95%), exhibited ABTS radical scavenging rates above 50%. This study provides theoretical and technical foundations for the extraction and functional development of volatile oils from *W. villosa* leaves.

## 1. Introduction

Critical components of both traditional and modern pharmacopeias are plant-derived essential oils and bioactive compounds—particularly volatile oils (also termed essential or aromatic oils)—that exhibit distinct pharmacological properties, including anti-inflammatory, antimicrobial, and antioxidant activities [1]. These volatile liquids, extracted from plant fruits, flowers, leaves, and roots, predominantly comprise low-molecular-weight compounds, such as alcohols, aldehydes, ketones, phenols, and terpenes [2]. Typically, pale yellow or colorless, they are often regarded as ‘liquid gold’ due to their high economic and therapeutic value [3]. Amid increasing evidence of potential adverse effects associated with synthetic antioxidants [4], plant-derived essential oils have garnered significant attention in pharmaceutical, cosmetic, and food industries owing to their potent antioxidant capacity.

Current extraction methods for volatile oils primarily include solvent extraction, hydrodistillation (HD), ultrasound-assisted extraction, microwave-assisted extraction, and supercritical CO_2_ extraction. Among these, HD remains the most widely used technique due to its operational simplicity, cost-effectiveness, and reasonable efficiency, despite limitations such as high energy consumption and prolonged extraction duration [5]. Consequently, optimizing solvent systems, developing green extraction technologies, enhancing extraction efficiency, minimizing energy requirements, and producing high-quality volatile oils have become critical research priorities. Enzyme-assisted extraction, as an eco-friendly alternative, improves the release of intracellular compounds through the selective degradation of plant cell wall cellulose by cellulase. This approach offers notable advantages, including enhanced efficiency, environmental sustainability, and better preservation of bioactive components’ pharmacological properties [6,7]. For example, cellulase-assisted extraction demonstrated 1.46-fold greater efficiency compared to conventional methods in *Curcuma phaeocaulis* Valeton volatile oil production [8]. Similarly, Li et al. [9] reported a 4.1% yield for *Rosmarinus officinalis* L. essential oil using enzymatic methods, representing a 53% improvement over conventional HD yields. These studies collectively underscore the significant potential of enzyme-assisted extraction technology in volatile oil production.

*Wurfbainia villosa* (Lour.) Škorničk. & A.D.Poulsen *(W. villosa*), a perennial herb of the Zingiberaceae family, is an important traditional Chinese medicinal plant indigenous to Yangchun, Guangdong Province, primarily distributed in Guangdong, Yunnan, and Hainan. With a medicinal history exceeding 1300 years [10], it produces bioactive compounds including monoterpenes, sesquiterpenes, and flavonoids [11], which exhibit gastrointestinal protection, hypoglycemic effects, and anti-inflammatory activities [12,13,14,15,16,17]. Studies have shown that *W. villosa* essential oil enhances wound healing in rats, improves antioxidant status, and inhibits non-alcoholic fatty liver disease progression [18,19]. Its volatile oil contains characteristic compounds (e.g., bornyl acetate, β-pinene) [20,21,22], with both fruits and leaves providing therapeutically relevant components [23,24,25]. Current research focuses on fruit-derived oils, while the utilization of stems and leaves remains underexplored despite their potential as medicinal resources.

This study optimized the cellulase-assisted hydrodistillation (cellulase-HD) extraction process and systematically compared the chemical composition differences between cellulase-HD and conventional HD-derived volatile oils. GC-MS analysis was employed to characterize volatile components and identify distinctive markers from different extraction methods. The antioxidant activity of *W. villosa* leaf volatile oil was evaluated using DPPH and ABTS radical scavenging assays, with a particular focus on screening and identifying active components in cellulase-HD extracts. The research elucidated the molecular mechanisms underlying the antioxidant activity of these volatile oil components and established structure–activity relationships between their chemical structures and antioxidant performance. The findings provide both a theoretical foundation and technical support for the extraction and functional development of *W. villosa* leaf volatile oils.

## 2. Results and Discussion

### 2.1. Single-Factor Screening

#### 2.1.1. The Effect of the Enzyme Dosage on the Extraction Yield of Volatile Oil from *W. villosa* Leaves

**Figure 1:**
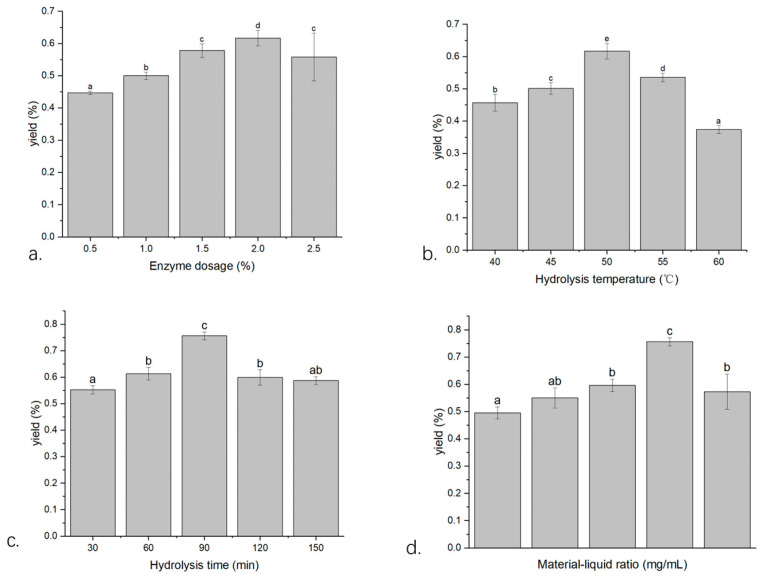
Effects of enzymatic hydrolysis conditions on the extraction yield of *W. villosa* leaf volatile oil: ((**a**) enzyme dosage; (**b**) hydrolysis temperature; (**c**) hydrolysis time; (**d**) material/liquid ratio). Note: Different lowercase letters indicate statistically significant differences (*p* < 0.05) in the extraction yield of *W. villosa* leaf volatile oil among different treatment levels, while the same letters denote non-significant differences (*p* > 0.05).

As shown in Figure 1a, within the enzyme dosage range of 0.5–2.0%, the extraction yield of volatile oil from *W. villosa* leaves gradually increased with increasing enzyme amount. When the enzyme dosage reached 2.0%, the substrate was fully hydrolyzed, and the extraction yield peaked at 0.617%. However, when the enzyme dosage increased to 2.5%, the extraction yields significantly decreased by 9.56% (*p* < 0.05) compared to that at 2.0%. This phenomenon may be attributed to the formation of an adhesion layer composed of enzymes and partially hydrolyzed cellulose on the surface of *W. villosa* leaves at 2.0% enzyme dosage, which hindered direct contact between volatile oil and the extraction solvent [26], thereby inhibiting the dissolution of volatile oil. Therefore, 2.0% was determined as the optimal enzyme dosage.

#### 2.1.2. The Effect of Enzymatic Hydrolysis Temperature on the Extraction Yield of Volatile Oil from *W. villosa* Leaves

As shown in Figure 1b, within the temperature range of 40–60 °C, the extraction yield of volatile oil from *W. villosa* leaves first increased and then decreased. At temperatures below 50 °C, cellulase activity was low, resulting in poor hydrolysis efficiency. However, as the temperature increased, enzyme activity improved, and the collision probability between enzymes and the substrate increased [27], enhancing cell wall decomposition and, thereby, increasing the extraction yield. At 50 °C, the extraction yield peaked at 0.617%. When the temperature exceeded 55 °C, the yield decreased to 0.534%, a reduction of 13.45% (*p* < 0.05) compared to that at 50 °C. At 60 °C, the yield further decreased significantly (*p* < 0.05), with a decline of 39.38%. This is because temperature has a dual effect on cellulase activity; beyond 50 °C, enzyme activity declines, leading to reduced extraction yield. Therefore, 50 °C was determined as the optimal enzymatic hydrolysis temperature.

#### 2.1.3. The Effect of Enzymatic Hydrolysis Time on the Extraction Yield of Volatile Oil from *W. villosa* Leaves

As shown in Figure 1c, within the hydrolysis time range of 30–90 min, the extraction yield of volatile oil from *W. villosa* leaves increased with prolonged hydrolysis time. When the hydrolysis time was less than 90 min, cellulose was not fully decomposed. At 90 min, the enzymatic reaction was complete, and the extraction yield peaked at 0.756%. However, when the hydrolysis time was extended to 120 min and 150 min, the yields decreased to 0.599% and 0.587%, respectively, representing significant declines of 20.76% (*p* < 0.05) and 22.35% (*p* < 0.05) compared to that at 90 min. This phenomenon may be due to the reduction of substrate over time and the inhibitory effect of hydrolysis products on the reaction [28], leading to decreased yields after 90 min. Therefore, 90 min was determined as the optimal enzymatic hydrolysis time.

#### 2.1.4. The Effect of Material/Liquid Ratio on the Extraction Yield of Volatile Oil from *W. villosa* Leaves

As shown in Figure 1d, within the material/liquid ratio range of 1:4–1:12, the extraction yield of volatile oil from *W. villosa* leaves first increased and then decreased. When the ratio was below 1:10, insufficient solvent volume resulted in incomplete extraction of volatile oil and inadequate binding between cellulase and the substrate [29], leading to lower yields. As the solvent volume increased, the mass concentration gradient between intracellular and extracellular substances increased, promoting the dissolution of volatile oil components. At a ratio of 1:10, the extraction yield peaked at 0.756%. However, when the ratio increased to 1:12, the yield decreased to 0.573%, a significant reduction of 24.21% (*p* < 0.05) compared to that at 1:10. This is because an excessively high material/liquid ratio increases water content in the system, diluting enzyme concentration and inhibiting the enzymatic reaction, thereby reducing the extraction yield [30]. Therefore, the optimal material/liquid ratio was determined to be 1:10.

### 2.2. Optimization of Cellulase-HD Process for W. villosa Leaf Volatile Oil Extraction Using a Box–Behnken Design

Using enzyme dosage (A), hydrolysis temperature (B), hydrolysis time (C), and material/liquid ratio (D) as experimental factors, with the extraction yield of *W. villosa* leaf volatile oil as the response value, a 4-factor 3-level optimization experiment was designed based on the Box–Behnken methodology. The experimental design and corresponding results are presented in Table 1.

Regression analysis was performed using Design-Expert 13 software on the extraction yield of *W. villosa* volatile oil with respect to enzyme dosage, hydrolysis temperature, hydrolysis time, and material/liquid ratio in Table 2, yielding the quadratic polynomial regression equation: Y = 0.7053 + 0.0142A − 0.0001B−0.0091C + 0.0230D − 0.0488AB − 0.0682AC + 0.0250AD + 0.0275BC + 0.0150BD − 0.0605CD − 0.0785A^2^ − 0.1206B^2^ − 0.0549C^2^ − 0.1000D^2^, where Y is the extraction yield of *W. villosa* leaf volatile oil (%); A, B, C, and D represent enzyme dosage (%), hydrolysis temperature (°C), hydrolysis time (min), and material/liquid ratio (g/mL), respectively.

The significance test and ANOVA results of the quadratic regression model are presented in Table 2. The model’s F-value of 34.15 with *p* < 0.0001 indicates that the regression model is highly significant. The lack-of-fit test yielded a *p*-value of 0.4005 (>0.05), indicating non-significance and confirming the model’s predictive validity. Additionally, the model’s correlation coefficient R^2^ of 0.9577 indicates a strong fit between response values and independent variables, with a significant linear relationship, making it suitable for theoretical predictions. The adjusted R^2^ (R^2^Adj) of 0.9469 further verifies the excellent agreement between experimental values and the regression equation [31], effectively explaining response variations.

Linear analysis of regression coefficients reveals that D, AB, AC, CD, A^2^, B^2^, C^2^, and D^2^ exert highly significant effects on volatile oil yield, while A, BC, and BD show significant influence. The descending order of enzymatic condition effects on *W. villosa* leaf volatile oil yield is as follows: material/liquid ratio > enzyme dosage > hydrolysis time > hydrolysis temperature. Therefore, this model can reliably optimize the cellulase-HD process for extracting volatile oil from *W. villosa* leaves.

**Figure 2:**
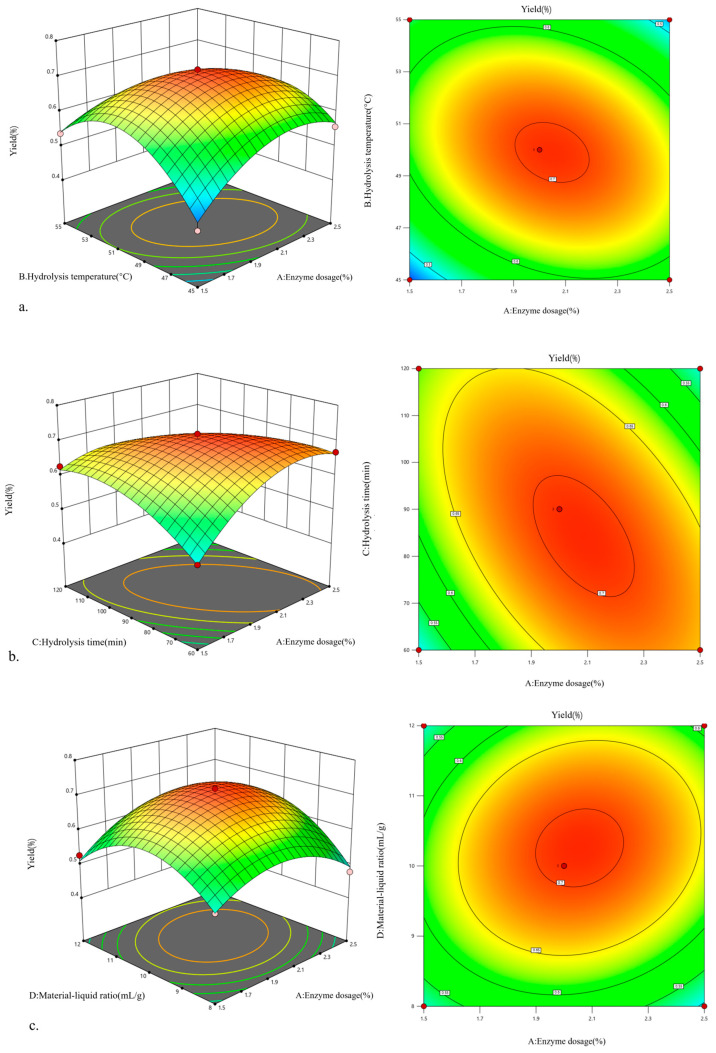

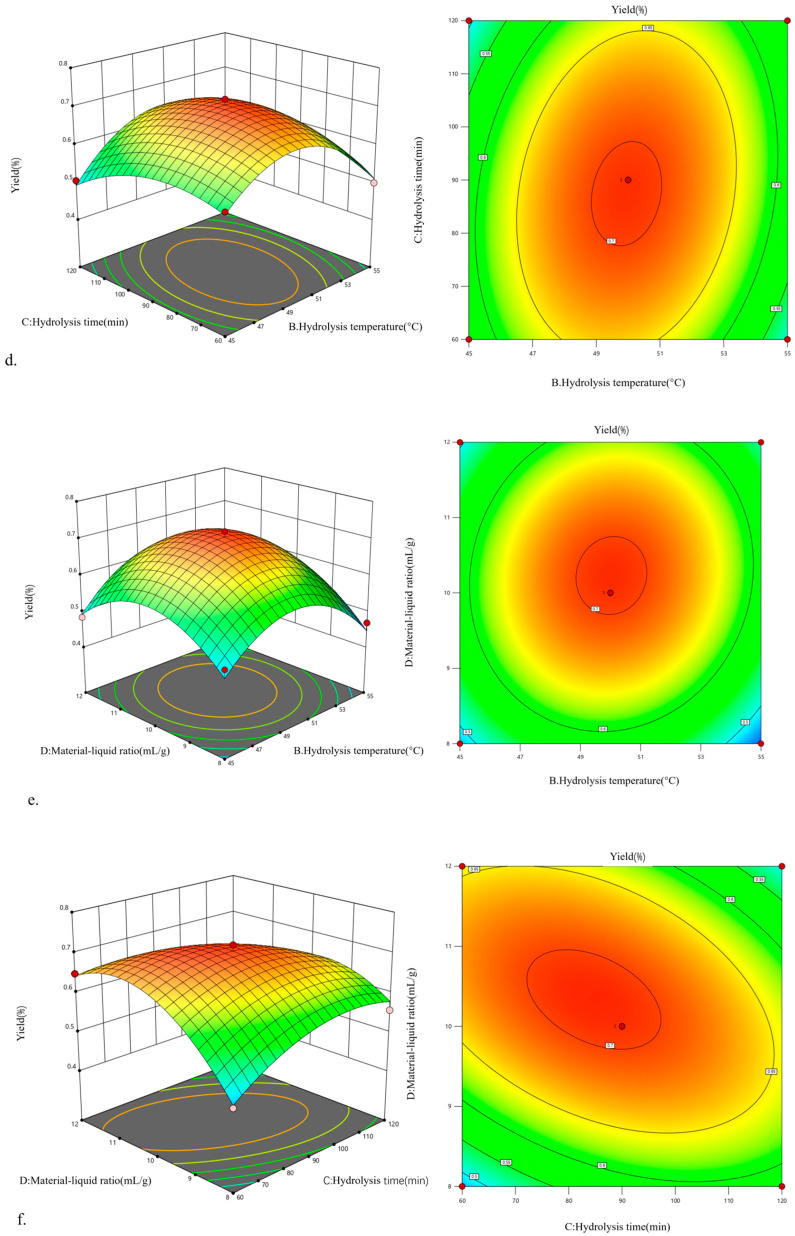
Response surface and contour plots for the interactions of factors for cellulase-HD ((**a**) interaction between A and B; (**b**) interaction between A and C; (**c**) interaction between A and D; (**d**) interaction between B and C; (**e**) interaction between B and D; (**f**) interaction between C and D).

Figure 2 presents the contour plots and response surface plots illustrating the pairwise interactions among four factors: enzyme dosage, hydrolysis temperature, hydrolysis time, and material/liquid ratio. The response surface and contour plots visually demonstrate each factor’s degree of influence. When the response surface of two interacting factors shows steeper curvature with more elliptical and denser contour lines [31,32], it indicates more significant effects on the extraction yield of *W. villosa* leaf volatile oil.

As shown in subplots (a), (b), (c), (d), and (f), the steep curvature of response surfaces between enzyme dosage and hydrolysis temperature, enzyme dosage and hydrolysis time, enzyme dosage and material/liquid ratio, hydrolysis temperature and hydrolysis time, and hydrolysis time and material/liquid ratio confirms significant interactive effects on volatile oil yield. However, subplot (e) reveals a relatively flat response surface for the interaction between hydrolysis temperature and material/liquid ratio, suggesting an insignificant influence on the extraction yield. These observations align perfectly with the ANOVA results of the regression model.

### 2.3. Optimization and Validation of Hydrolysis Conditions

Through Design-Expert 13 software analysis, the optimal process parameters were determined: enzyme dosage 2.213%, hydrolysis temperature 49.347 °C, hydrolysis time 73.131 min, and material/liquid ratio 1:10.657 (mg/mL), with a predicted volatile oil yield of 0.715%. For practical operation, the optimal conditions were slightly adjusted: enzyme dosage 2.2%, hydrolysis temperature 49 °C, hydrolysis time 73 min, and material/liquid ratio 1:10.7 (mg/mL).

Triplicate verification experiments yielded an average extraction rate of 0.772%, with only 0.056% standard deviation from the theoretical value, demonstrating excellent agreement between experimental and predicted results. Compared with the maximum yield (0.756%) obtained in single-factor experiments, the optimized conditions achieved a 0.016% improvement. More remarkably, the yield increased by 1.3-fold compared with HD (0.588%), showing a 90.67% increase compared to the steam distillation yield (1.18‰) reported by Zhang [23]. These results confirm that the response surface methodology provides accurate and reliable optimization of cellulase-HD parameters for *W. villosa* leaf volatile oil extraction, demonstrating a high practical value.

### 2.4. Microstructure Analysis of W. villosa Leaves

**Figure 3:**
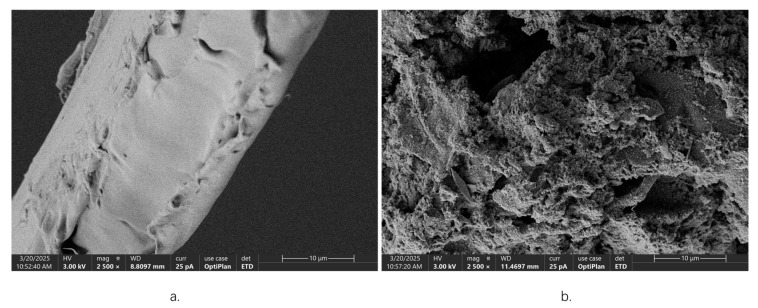
SEM micrographs of *W. villosa* leaves at 2500× magnification after different pretreatments ((**a**) *W. villosa* leaves without treatment; (**b**) cellulase treatment of *W. villosa* leaves).

Scanning electron microscopy (SEM) was employed to examine the microstructural alterations in *W. villosa* leaves following different pretreatment methods, with representative micrographs presented in Figure 3. At 2500× magnification, untreated leaf specimens displayed intact cellular morphology featuring smooth surfaces and tightly interconnected cell walls, presumably due to the presence of natural cuticular waxes and protective tissue layers. In marked contrast, cellulase-pretreated leaves exhibited substantial surface modifications, including increased roughness, irregular topography, and structural discontinuity. These morphological changes resulted from the enzymatic degradation of cell wall structural polysaccharides (cellulose and hemicellulose) by cellulase [33], which partially disrupted the cell wall integrity. The observed structural modifications significantly enhanced the permeability of cellular compartments, thereby facilitating the release and subsequent extraction of volatile oil constituents and accounting for the improved extraction efficiency.

### 2.5. Chemical Composition Analysis of W. villosa Leaf Volatile Oil

The chemical profiles of volatile oils extracted from *W. villosa* leaves via HD and cellulase-HD were comparatively analyzed using gas chromatography–mass spectrometry (GC-MS). As summarized in Table 3 GC-MS analysis identified a total of 65 volatile compounds, comprising 49 and 54 constituents in HD and cellulase-HD extracts, respectively. Intersection analysis revealed 39 shared compounds between the two methods, while HD and cellulase-HD exhibited 10 and 15 unique components, respectively. Notably, the cellulase-HD method demonstrated significantly enhanced extraction efficiency, yielding 10.2% more volatile constituents than conventional HD. This improvement can be attributed to the enzymatic biodegradation of cellular structural components by cellulase, which facilitated the liberation of small-molecule bioactive compounds from the leaf matrix.

The volatile oil extracted from *W. villosa* leaves by HD was predominantly composed of β-phellandrene (41.881%), β-myrcene (8.656%), β-pinene (4.743%), limonene (8.444%), γ-terpinene (6.601%), 4-terpineol (4.974%), and α-terpineol (2.751%). In contrast, the cellulase-HD extract contained camphene (2.897%), β-phellandrene (10.634%), limonene (13.352%), γ-terpinene (14.981%), 4-carene (5.572%), 4-terpineol (10.145%), α-terpineol (8.085%), borneol (2.333%), myrtenol (3.073%), myrtenal (2.740%), methyl myrtenate (2.727%), and o-cymene (4.038%) as major components. Comparative analysis revealed that the cellulase-HD method significantly reduced β-phellandrene content while substantially increasing the yields of limonene (13.352%), γ-terpinene (14.981%), and 4-terpineol (10.145%) compared to conventional HD.

The current findings differ substantially from those reported by Zhang et al. [23], who identified 3,7,7-trimethyl-1,3,5-cycloheptatriene, β-pinene, linalyl acetate, β-caryophyllene, and trans-Z-α-bergamotene as major components. However, certain similarities were observed in Huang et al. [24]’s study, which reported pseudolimonene (41.12%), 3-carene (24.17%), D-limonene (3.25%), and myrtenol (3.18%) as predominant constituents in *W. villosa* leaves. These compositional variations likely stem from differences in plant species, geographical origin, and extraction methodologies.

**Figure 4:**
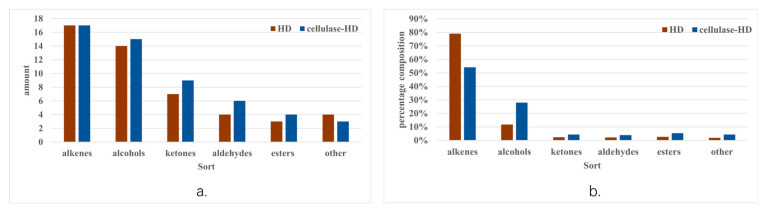
(**a**,**b**) Differences in the composition and content of volatile oil obtained from HD and cellulase-HD.

As shown in Figure 4, comparative analysis of volatile oils extracted by HD and cellulase-HD revealed distinct compositional profiles. Both methods yielded 17 terpene compounds, while the number of alcohols (14 vs. 15), ketones (7 vs. 9), aldehydes (4 vs. 6), esters (3 vs. 4), and other constituents (4 vs. 3) differed between the two techniques. Notably, cellulase-HD enabled the identification of significantly more volatile components than conventional HD. Quantitative analysis demonstrated substantial differences in chemical composition between the extraction methods. The terpene content decreased markedly from 79.099% (HD) to 54.184% (cellulase-HD), whereas alcohol content showed a significant increase from 11.724% to 27.956%. Furthermore, the relative abundances of ketones, aldehydes, and esters exhibited respective increases from 2.381%, 2.251%, and 2.687% (HD) to 4.374%, 3.915%, and 5.313% (cellulase-HD). The proportion of other compounds also rose from 1.859% to 4.257%. Of particular significance, previous studies have established that oxygenated compounds (including alcohols, ketones, aldehydes, and esters) represent the primary bioactive constituents in essential oils and play crucial roles in antioxidant activities [34]. The current findings demonstrate that extraction methodology significantly influences the chemical composition of *W. villosa* leaf volatile oils, which may directly correlate with variations in their biological activities.

### 2.6. Orthogonal Partial Least Squares Discriminant Analysis (OPLS-DA) Analysis of Volatile Oil Components from HD and Cellulase-HD Extracts

**Figure 5:**
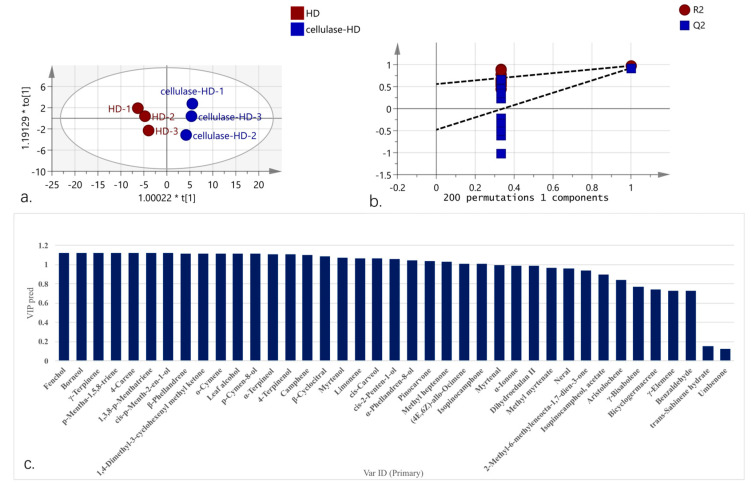
Orthogonal partial least squares discriminant analysis (OPLS-DA) analysis of volatile oil from *W. villosa* leaves obtained by HD and cellulase-HD ((**a**). score chart; (**b**). replacement test results; (**c**). VIP value).

To characterize compositional differences between extraction methods, we performed supervised Orthogonal partial least squares discriminant analysis (OPLS-DA), a multivariate analysis technique that enables dimensionality reduction and discriminant analysis under predefined classification conditions [35]. The model quality was assessed using three key parameters: R^2^X (explained variance of X-matrix), R^2^Y (explained variance of Y-matrix), and Q^2^ (predictive capability). Values approaching 1.0 indicate optimal model performance, with thresholds >0.4 generally considered acceptable when the R^2^-Q^2^ difference is minimal [36,37]. OPLS-DA model, constructed with extraction methods as independent variables and volatile components as dependent variables, demonstrated excellent performance (R^2^X = 0.926, R^2^Y = 0.973, Q^2^ = 0.921) following 200 permutation tests. These results confirm both model stability (R^2^X→1) and predictive reliability (Q^2^ > 0.8) [38]. The score plot (Figure 5a) revealed clear spatial separation between HD and cellulase-HD samples, confirming significant compositional alterations induced by enzymatic pretreatment. Permutation testing (n = 200; Figure 5b) validated model robustness, with negative Q^2^-Y-intercept confirming absence of overfitting.

Twenty-six differential components (VIP > 1, *p* < 0.05) were identified as characteristic markers (Figure 5c), ranked by contribution magnitude: fenchol, borneol, γ-terpinene, p-mentha-1,5,8-triene, 4-carene, 1,3,8-p-menthatriene, cis-p-menth-2-en-1-ol, β-phellandrene, 1,4-dimethyl-3-cyclohexenyl methyl ketone, o-cymene, leaf alcohol, p-Cymen-8-ol, α-terpineol, 4-terpinenol, Camphene, β-cyclocitral, myrtenol, limonene, cis-carveol, cis-2-penten-1-ol, α-phellandren-8-ol, pinocarvone, methyl heptanone, (4E,6Z)-allo-ocimene, and isopinocamphone. These markers effectively discriminate between HD and cellulase-HD derived volatile oils from *W. villosa* leaves.

### 2.7. Antioxidant Activity of W. villosa Leaf Volatile Oil

#### 2.7.1. DPPH Radical Scavenging Assay

**Figure 6:**
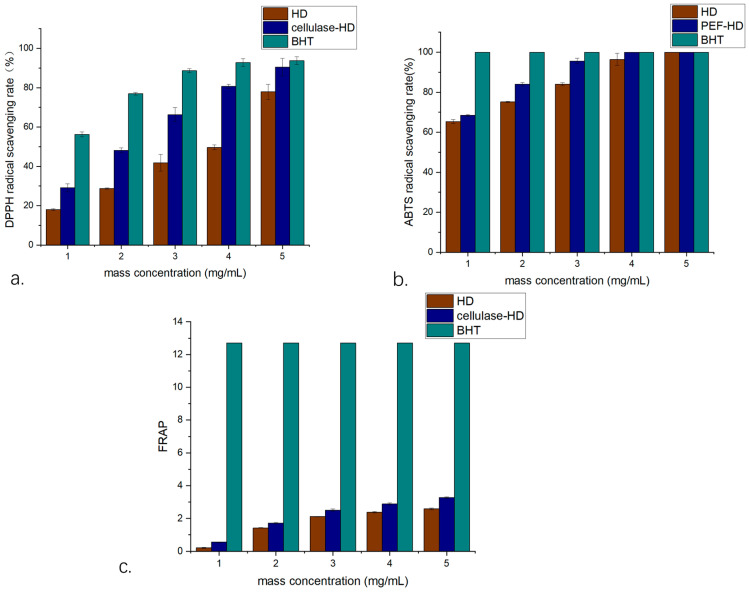
Antioxidant activity of volatile oil from *W. villosa* leaves ((**a**). DPPH radical scavenging rate; (**b**). radical scavenging rate of ABTS; (**c**). TAC).

The antioxidant capacity of volatile oils extracted via both methods was evaluated using DPPH radical scavenging assays (Figure 6a). Both cellulase-HD and HD extracts demonstrated concentration-dependent antioxidant activity within the tested range (1–5 mg/mL). Comparative analysis revealed significantly enhanced (*p* < 0.05) radical scavenging capacity in cellulase-HD extracts across all concentrations. At the maximum tested concentration (5 mg/mL), scavenging rates reached 77.94% ± 1.2% (HD) and 90.46% ± 0.8% (cellulase-HD), with corresponding IC50 values of 3.46 mg/mL (95% CI: 3.32–3.61) and 2.73 mg/mL (95% CI: 2.61–2.85), respectively. When normalized to the positive control (BHT), the scavenging capacities represented 83% and 96% of BHT’s activity at equivalent concentration (5 mg/mL), confirming the substantial antioxidant potential of both extracts.

#### 2.7.2. ABTS Radical Scavenging Assay

The ABTS radical scavenging capacity of both extracts displayed concentration-dependent enhancement within the 1–5 mg/mL range (Figure 6b). The HD extract achieved maximal scavenging (100%) at 5 mg/mL (IC50 = 2.10 mg/mL; 95% CI: 2.01–2.19), whereas the cellulase-HD extract reached complete neutralization at 3 mg/mL (IC50 = 1.87 mg/mL; 95% CI: 1.79–1.95). Comparative analysis revealed significantly stronger (*p* < 0.01) scavenging activity in CA-HD extracts across the 1–4 mg/mL concentration range. While the positive control (BHT, 1 mg/mL) demonstrated complete (100%) scavenging, both *W. villosa* volatile oil extracts exhibited substantial ABTS radical neutralization capacity, with CA-HD showing particularly potent activity approaching the reference standard.

#### 2.7.3. Total Antioxidant Capacity (TAC)

The reducing power of volatile oils extracted by both methods was evaluated using FRAP assays across a concentration gradient (1–5 mg/mL) (Figure 6c). Both HD and CA-HD extracts demonstrated concentration-dependent increases in antioxidant capacity, with cellulase-HD extracts exhibiting significantly greater (*p* < 0.05) reducing power at all equivalent concentrations. At 5 mg/mL, FRAP values reached 3.20 ± 0.15 and 2.58 ± 0.12 for cellulase-HD and HD extracts, respectively, with corresponding IC50 values of 0.41 (95% CI: 0.38–0.44) and 0.726 (95% CI: 0.69–0.76) FRAP units. Although the antioxidant capacity remained substantially lower than the positive control (BHT, 12.71 ± 0.32 FRAP units), both extracts demonstrated notable reducing activity among plant-derived antioxidants. These results indicate that cellulase-assisted extraction enhances the liberation of bioactive compounds, consequently improving the antioxidant potential of the volatile oils. The findings provide empirical support for utilizing *W. villosa* leaf resources in natural antioxidant development.

Antioxidant capacity is defined as the ability of compounds to scavenge free radicals and counteract oxidative stress, which arises from an imbalance between reactive oxygen species (ROS) production and endogenous antioxidant defenses. ROS comprise highly reactive molecules with unpaired electrons that can induce oxidative damage through diverse chemical pathways. Comprehensive antioxidant evaluation requires multiple standardized assays, as different methods probe distinct mechanistic pathways: the DPPH assay primarily measures hydrogen atom transfer capacity, whereas the ABTS assay predominantly assesses single electron transfer activity [39]. The enhanced antioxidant activity observed in cellulase-HD extracts of *W. villosa* leaves correlates with their increased concentration of oxygenated terpenoids, particularly 4-terpineol and α-terpineol. Scientific evidence confirms that 4-terpineol mitigates oxidative stress-induced cellular damage [40], while α-terpineol effectively suppresses superoxide generation and demonstrates superior ABTS radical scavenging capacity [39,41]. Furthermore, endo-borneol has been documented to enhance oxidative stress resistance and potentiate the efficacy of therapeutic antioxidants [42,43]. The overall antioxidant performance may also be influenced by synergistic or antagonistic interactions among volatile oil constituents. To systematically identify active components, we developed an integrated antioxidant-GC-MS approach. This method enables rapid screening of antioxidant compounds by quantifying chromatographic peak area variations before and after radical reactions, thereby establishing direct structure–activity relationships for individual volatile oil constituents.

### 2.8. Identification of Antioxidant Active Ingredients

#### 2.8.1. Antioxidant Components of DPPH Free Radicals

**Figure 7:**
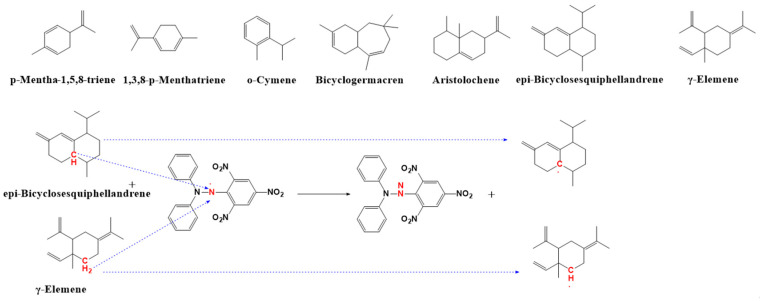
The reaction mechanism of scavenging DPPH free radicals.

Analysis of Table 4 data revealed that 34 volatile components exhibited varying degrees of DPPH radical scavenging activity after the reaction. Seven compounds demonstrated particularly strong scavenging capacity (>50%), ranked in descending order of efficacy: γ-elemene (78.91%), epi-bicyclosesquiphellandrene (71.51%), aristolochene (58.36%), bicyclogermacrene (55.72%), o-cymene (55.03%), p-mentha-1,5,8-triene (54.42%), and p-mentha-1,3,8-triene (53.91%). Notably, epi-bicyclosesquiphellandrene and γ-elemene displayed exceptional DPPH radical scavenging capabilities. Structure–activity relationship analysis indicated that all highly active compounds contain carbon–carbon double bonds (C=C) as a common structural feature. The C=C bonds activate adjacent α-hydrogens (α-H), facilitating C-H bond cleavage and hydrogen atom transfer processes [44]. γ-Elemene contains multiple C=C bonds, with its allylic hydrogens (allylic H) and tertiary hydrogens (tertiary H) exhibiting enhanced reactivity due to stabilization effects from adjacent double bonds and carbocation formation tendency, accounting for its superior radical scavenging capacity; epi-bicyclosesquiphellandrene, as a bicyclic sesquiterpene, demonstrates increased reactivity in its bridge-ring structure due to the combined effects of ring strain and conjugated double bonds [44] on its multiple tertiary and allylic hydrogens. The radical scavenging mechanisms of these compounds are detailed in Figure 7.

#### 2.8.2. Antioxidant Components of ABTS Free Radicals

**Figure 8:**
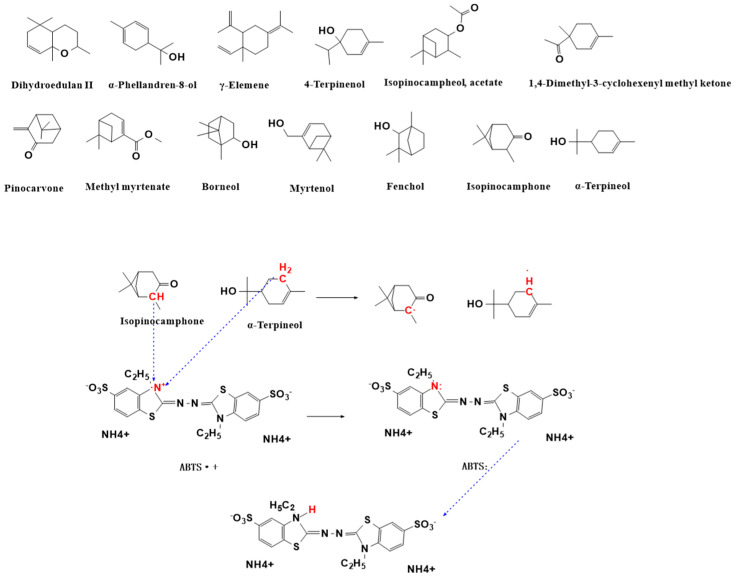
The radical scavenging mechanism of ABTS.

In the ABTS radical scavenging assay, 13 compounds demonstrated significant scavenging activity (>50%), ranked in the descending order of efficacy: α-terpineol (66.95%), isopinocamphone (66.58%), 1,4-dimethyl-3-cyclohexenyl methyl ketone (66.55%), myrtenal (65.57%), endo-borneol (65.04%), methyl myrtenate (65.03%), pinocarvone (64.85%), fenchol (64.80%), isopinocampheyl acetate (62.94%), 4-terpineol (62.77%), γ-elemene (57.67%), α-phellandren-8-ol (55.96%), and dihydrosyringin II (54.55%). Notably, these active compounds were predominantly oxygenated terpenoids. Mechanistic studies revealed that ABTS radical scavenging primarily involves the following pathways: Oxygen anions donate single electrons to pair with unpaired electrons on ABTS cation radicals [45]. Ionized hydrogen ions combine with nitrogen’s lone electron pairs to form stable ABTSH products. The scavenging mechanism principally occurs through a proton-loss-prioritized electron transfer process [46]. Specifically, the process is as follows: In α-terpineol, the allylic hydrogen exhibits weakened C-H bonds due to synergistic effects between the adjacent hydroxyl (-OH) group and conjugated double bonds. The hydroxyl group may participate in the reaction via proton-coupled electron transfer (PCET), primarily reducing ABTS+ through single electron transfer or hydrogen atom transfer mechanisms. In isopinocamphone, the α-carbon hydrogen (α-C-H) adjacent to the carbonyl group is activated by the electron-withdrawing effect of the carbonyl (C=O), making it more susceptible to radical abstraction, predominantly reducing ABTS+· through the hydrogen atom transfer. These findings are consistent with previous reports: Ran et al. [47] demonstrated that γ-elemene exhibits remarkable ABTS radical scavenging capacity (67.21%). The detailed mechanisms of these compounds are illustrated in Figure 8.

## 3. Materials and Methods

### 3.1. Materials and Ecological Characteristics

Plant material: Fresh leaves of *W. villosa* Lour. were collected on 16 October 2024, from Chenwu Village, Chuncheng Sub-district, Yangchun City, Guangdong Province, and stored at −20 °C at the Yangchun *W. villosa* Superior Resource Cultivation Base.

Ecological characteristics: *W. villosa*, a tropical/subtropical plant, is native to areas between 111°16′27″–112°09′22″ E longitude and 21°50′36″–22°41′01″ N latitude. It typically grows in shaded valleys or along streams at elevations of 100–150 m, preferring warm, humid environments with diffused sunlight [48]. The plant thrives in well-drained, fertile, moist soil characterized by yellow clay subsoil and a humus-rich top layer (pH 4.8–5.6). Locally known as “stone-flower land,” this optimal soil consists of black sandy loam mixed with small gravel. Fertilization is generally unnecessary as it may inhibit flowering. Winter maintenance includes pruning, weeding, a single fertilization, and soil mounding around the base. During flowering, light watering facilitates pollen transfer to the micropyle for improved fruit set, while excessive watering should be avoided to prevent flower damage.

### 3.2. Chemicals and Reagents

n-Hexane (chromatographic grade, ≥99%); 2,6-Di-tert-butyl-4-methylpenol(BHT, AR, >99%); CH_3_CH_2_OH (AR, >99.5%); FeCl_3_·_6_H_2_O (AR, >99%); Na_2_SO_4_ (AR, >99%); K_2_S_2_O_8_ (AR, >99.5%); acetate buffer; Fe_2_(SO_4_)_3_; acetic acid buffer, phosphoric acid buffer; cellulase (1000 U/g) were provided by Shanghai Macklin Biochemical Co., Ltd. (Shanghai, China). 1,1-Diphenyl-2-picrylhydrazyl (DPPH, ≥98%) was acquired from Shanghai Yuanye Bio-Technology Co., Ltd. (Shanghai, China). 2,2′-Azino-bis(3-ethylbenzothiazoline-6-sulfonic acid) diammonium salt (ABTS, ≥98%) was supplied by Shanghai Macklin Biochemical Co., Ltd. (Shanghai, China). 2,4,6-Tris(2-pyridyl)-s-triazine (TPTZ, ≥98%) was purchased from Hefei Bomei Biotechnology Co., Ltd. (Hefei, China).

### 3.3. Instruments and Equipment

A 5 mL volatile oil extractor (Beijing Glass Instrument Factory, Beijing, China); a 2000 mL borosilicate round-bottom flask (Shuniu Brand, China); a 30 cm spherical condenser with 24/29 joints (Shanghai Yarong, Shanghai, China); TC3K-H electronic balance (3 kg capacity, 0.1 g accuracy, Changshu Shuangjie Testing Instrument Factory, Changshu, China); ME204 analytical balance (220 g capacity, 0.1 mg accuracy, Mettler-Toledo, Shanghai, China); 2000 mL SKM digital heating mantle (RT~450 °C, ±1 °C, Juancheng Hualu, China); JYL-C23 juice blender (900 W, 16,000 rpm, Jiuyang Co., Ltd., China); HJ-M4 magnetic stirring water bath (RT~100 °C, Shanghai Hetian Scientific Instrument, Shanghai, China); FE28 pH meter (±0.01 pH accuracy, Mettler-Toledo, Shanghai, China); Apreo 2S scanning electron microscope (0.8 nm resolution, 0.2–30 kV, Thermo Fisher Scientific, Guangzhou, China); Agilent 7000D gas chromatography–mass spectrometer equipped with DB-WAX column (30 m × 0.25 mm × 0.25 μm, m/z 10–1500, 1 μL injection volume, 50:1 split ratio, Agilent Technologies, China).

### 3.4. Extraction of Volatile Oil from W. villosa Leaves

#### 3.4.1. Volatile Oil Extraction from *W. villosa* leaves by Hydrodistillation (HD)

The volatile oil extraction from *W. villosa* leaves was performed in accordance with the volatile oil extraction method in General Chapter 2204 of the Chinese Pharmacopoeia (2020 Edition) [49]. The fresh leaves of *W. villosa* were cut into 3 cm sections, and 50.0 g was accurately weighed, mixed with 500 mL distilled water, and blended into a pulp. The mixture was placed in a 2000 mL round-bottomed flask and was soaked for 1 h. The volatile oil extractor was installed, an appropriate amount of distilled water was added, and the spherical condenser was connected. After heating at 160 °C for 4 h, the upper volatile oil layer was collected and dehydrated with anhydrous sodium sulfate. Finally, the volatile oil from *W. villosa* leaves was obtained. The volatile oil yield was calculated using Formula (1):

Y (%) = m/M × 100%(1)

where Y was the volatile oil extraction yield (%), m was the mass of volatile oil (g), and M was the dry weight of *W. villosa* leaves (g).

#### 3.4.2. Cellulase-Assisted Hydrodistillation (Cellulase-HD)

Fresh *W. villosa* leaves were precisely sectioned into 3 cm segments. Exactly 50.0 g of the prepared leaf material was homogenized with 500 mL of distilled water using a laboratory homogenizer, and the resulting slurry was transferred to a 1000 mL beaker. Cellulase was then added to initiate enzymatic hydrolysis. The reaction system pH was maintained at 5.0 using phosphate buffer solution. The enzymatic hydrolysis was carried out under controlled temperature and time conditions. Following hydrolysis completion, the essential oil was extracted according to the methodology detailed in Section 3.4.1.

##### Single-Factor Screening

The method of Tan [50] is referenced, with slight modifications. The single-factor experiment examined four parameters at varying levels: enzyme dosage (0.5–2.5% *w*/*w* of sample mass), hydrolysis temperature (35–55 °C), hydrolysis duration (30–150 min), and solid-to-liquid ratio (1:4–1:12 g/mL). Each factor’s individual effect on volatile oil yield from *W. villosa* leaves was systematically evaluated. Volatile oil yield was quantified using the method described in Section 3.4.2.

##### Box–Behnken Experimental Design

Following single-factor analysis, four key variables were selected for optimization: enzyme dosage, hydrolysis temperature, duration, and material/liquid ratio. A Box–Behnken design was implemented, featuring four factors at three levels, with volatile oil yield as the response variable (Table 5). This design was informed by preliminary single-factor experiments.

### 3.5. Chemical Composition Analysis of Volatile Oil from W. villosa Leaves

The method of Huang [24] is referenced, with slight modifications. GC temperature program: column: DB-WAX, injection port 250 °C, flow rate 1.2 mL/min, and splitless headspace injection. Temperature program: initial temperature 40 °C held for 3 min, ramped at 3 °C/min to 130 °C (hold 3 min), 6 °C/min to 190 °C (hold 3 min), and 10 °C/min to 240 °C (hold 3 min).

MS conditions: electron bombardment ion source (EI), ionization energy 70 eV, ion source temperature 230 °C, full scanning mode, mass range 50–500 mz, and solvent delay time 3 min.

### 3.6. Scanning Electron Microscope (SEM) Analysis

The method of Pan [32] is referenced, with slight modifications. SEM was conducted on untreated *W. villosa* leaves, cellulase-hydrolyzed leaves, followed by gold sputter coating. Microstructure observation was performed at 2500× magnification, at 3 kV, with 10–11 mm working distance.

### 3.7. The Determination of the Antioxidant Capacity of Volatile Oil from W. villosa Leaves

#### 3.7.1. DPPH Radical Scavenging Activity Assay

The DPPH radical scavenging assay was performed according to Sun et al. [51]. Ethanol solutions of *W. villosa* leaf volatile oil were prepared at 1–5 mg/mL concentrations in anhydrous ethanol. A 2 mL aliquot of test solution was mixed with 2 mL anhydrous ethanol and 2 mL 0.05 mmol/L DPPH ethanol solution, then incubated in the dark for 30 min. Absorbance was measured at 517 nm (A_1_). For blank control (A_0_), 2 mL of anhydrous ethanol replaced the DPPH solution. Another 2 mL test solution was mixed with 2 mL of anhydrous ethanol. After 30 min of dark incubation, absorbance was measured (A_2_). All assays were performed in triplicate using BHT (1–5 mg/mL) as a positive control. The radical scavenging rate of DPPH was calculated according to the following formula:

I (%) = 1 − (A_1_ − A_0_)/A_2_ × 100%

where I is the DPPH radical scavenging rate (%); A_0_ is the blank group absorbance; A_1_ is the sample group absorbance; and A_2_ is the absorbance with ethanol replacing DPPH solution.

#### 3.7.2. ABTS Radical Scavenging Activity Assay

The ABTS radical scavenging assay was performed according to Li et al. [52]. ABTS working solution preparation: Equal volumes (50 mL each) of 1.48 mmol/L ABTS and 2.6 mmol/L potassium persulfate were mixed, and they reacted in the dark for 12 h, diluted with ethanol to an absorbance of 0.70 ± 0.05 at 734 nm (A_0_). Ethanol solutions of *W. villosa* leaf volatile oil were prepared at 1–5 mg/mL concentrations. A 2 mL test solution aliquot was mixed with 2 mL ABTS working solution, vortexed, and reacted for 15 min. Absorbance was measured at 734 nm (A_1_); the experiment was performed in triplicate and used BHT (1–5 mg/mL) as a positive control. The clearance rate was calculated by the following formula:

I (%) = (A_0_ − A_1_)/A_0_ × 100%

where I is ABTS radical scavenging rate (%); A_0_ is ABTS working solution absorbance; and A_1_ is sample group absorbance.

#### 3.7.3. Total Antioxidant Capacity Assay

The total antioxidant capacity was determined according to Gao et al. [53]. FRAP solution preparation: 0.3 mol/L acetate buffer (pH 3.5), 10 mmol/L TPTZ in HCl, and 20 mmol/L FeCl_3_·6H_2_O were mixed (10:1:1 v/v/v). All components were prepared fresh before use. Aliquots (125 μL) of 0.2–1.4 mmol/L FeSO_4_·7H_2_O solutions, with 4.5 mL of FRAP solution, were incubated at 37 °C for 30 min, and the absorbance was measured at 593 nm. Standard curve: Y = 0.7853 X + 0.0169 (R^2^ = 0.999).

To test samples (1 mL) of volatile oil–ethanol solutions (1–5 mg/mL), 4.5 mL of FRAP solution was added. Then, the prepared solution was incubated at 37 °C for 30 min, and the absorbance was measured at 593 nm. FRAP values were calculated using the standard curve, and BHT was used as the positive control. The total antioxidant capacity of *W. villosa* leaf volatile oil was expressed as FRAP values, where 1 FRAP value was 1 mmol/L FeSO_4_. Triplicate measurements were performed for each sample.

### 3.8. Screening of Antioxidant Components

#### 3.8.1. DPPH Radical Scavenging Activity Assessment

A 0.15 mL sample of *W. villosa* volatile oil was dissolved in 2 mL of absolute ethanol and mixed with 1 mL of 5 mmol/L DPPH-ethanol solution. The mixture was vortexed and incubated at 37 °C in the dark for 30 min. A control group was prepared by replacing the DPPH solution with an equal volume of absolute ethanol while maintaining identical conditions. Following the reaction, samples were analyzed according to the chromatographic conditions specified in Section 2.4. Antioxidant activity was evaluated by comparing chromatographic peak area variations before and after the radical reaction [54]. The radical scavenging rate was calculated using the following equation:

Scavenging rate (%) = (A_0_ − A_1_)/A_0_ × 100

where A_0_ is the peak area of each component in the control group (without radical reaction), and A_1_ is the peak area of each component in the test group (after radical reaction)

#### 3.8.2. ABTS Radical Scavenging Activity Assessment

A 0.15 mL volatile oil sample (dissolved in 2 mL absolute ethanol) was mixed with 1 mL ABTS working solution (prepared by mixing equal volumes of 3.7 mmol/L ABTS and 1.3 mol/L K_2_S_2_O_8_). The mixture was incubated at 37 °C in the dark for 30 min. The control group was prepared using absolute ethanol instead of the ABTS working solution. After chromatographic analysis under the conditions described in Section 2.4, the scavenging rate for each component was calculated according to the formula in Section 3.5. The variation in chromatographic peak areas was used to characterize the ABTS radical scavenging capacity of individual volatile oil components [55].

### 3.9. Data Processing and Analysis

All experiments were performed in triplicate, with results expressed as mean ± standard deviation. Volatile oil components were identified by comparing mass spectra with the NIST library (match factor ≥80), retention times (RT), and Kovats retention indices (KI). Quantitative analysis was performed using the peak area normalization method. Statistical analyses were conducted using SPSS software (version 26.0). Data visualization, including Venn diagrams, bar plots, and heatmaps, was performed using Origin 2021. OPLS-DA was carried out using SIMCA (version 14.1, Umetrics AB (Umeå, Sweden)) to evaluate compositional differences among volatile oil samples.

## 4. Conclusions

This study innovatively employed cellulase-HD for the extraction of volatile oils from *W. villosa* leaves. Through response surface methodology optimization, the optimal extraction parameters were determined as follows: enzyme dosage 2.2%, enzymatic hydrolysis temperature 49 °C, hydrolysis duration 73 min, and material/liquid ratio 1:10.7 (mg/mL). Under these conditions, the volatile oil yield reached 0.772%, representing a 31.29% improvement compared to HD, thus demonstrating significant technical advantages.

GC-MS analysis identified a total of 65 volatile compounds from both extraction methods, with 39 shared components. Significant differences in compositional profiles were observed between the two methods: the cellulase-HD extract contained substantially higher alcohol content (27.956%) than the HD extract (11.724%). OPLS-DA successfully differentiated the volatile oils obtained by different extraction methods and identified 25 key differential components, including characteristic compounds such as fenchol, borneol, and γ-elemene. Notably, terpenoids, as the primary active constituents, showed extraction method-dependent variations in their contents.

For antioxidant evaluation, this study established an “Antioxidant-GC-MS” structure–activity relationship model, enabling precise identification of chemical constituents and systematic assessment of their radical scavenging capacities in complex volatile oil samples. The research elucidated molecular-level mechanisms underlying the differential scavenging activities of various compounds against DPPH and ABTS radicals, clarifying the structure–function relationships of active components and providing experimental evidence for understanding the antioxidant mechanisms of *W. villosa* leaf volatile oils.

The findings confirm the potential of *W. villosa* leaf volatile oils as natural antioxidants while revealing the significant impact of extraction methods on both yield and chemical composition. This study not only provides theoretical guidance for industrial-scale extraction of *W. villosa* leaf volatile oils but also establishes a scientific foundation for their applications in food preservation and healthcare products.

## Figures and Tables

**Table 1:** The Box–Behnken design with experimental results for cellulase-HD.

No.	A: Enzyme Dosage (%)	B: Hydrolysis Temperature (°C)	C: Hydrolysis Time (min)	D: Material/Liquid Ratio (g/mL)	Yield (%)
1	2	50	120	12	0.495
2	2	50	60	12	0.649
3	2.5	45	90	10	0.557
4	1.5	45	90	10	0.421
5	2	55	90	12	0.515
6	2.5	50	90	12	0.591
7	2	50	90	10	0.692
8	2	55	60	10	0.500
9	2	55	90	8	0.469
10	2	45	90	8	0.499
11	2.5	50	60	10	0.668
12	2	45	60	10	0.574
13	2.5	50	90	8	0.478
14	1.5	50	60	10	0.499
15	2.5	50	120	10	0.523
16	1.5	50	120	10	0.627
17	2	50	60	8	0.469
18	2	55	120	10	0.542
19	1.5	50	90	8	0.514
20	2.5	55	90	10	0.478
21	1.5	50	90	12	0.527
22	2	50	120	8	0.557
23	2	45	120	10	0.506
24	2	45	90	12	0.485
25	2	50	90	10	0.704
26	2	50	90	10	0.720
27	1.5	55	90	10	0.537

**Table 2:** Response surface regression model and variance analysis results for cellulase-HD.

Source	Sum of Squares	df	Mean Square	F-Value	*p*-Value	
Model	0.1617	14	0.0115	34.15	<0.0001 **	significant
A—Enzyme dosage	0.0024	1	0.0024	7.12	0.0205 *	
B—Hydrolysis temperature	8.333 × 10^−8^	1	8.333E-08	0.0002	0.9877	
C—Hydrolysis time	0.0010	1	0.0010	2.93	0.1128	
D—Material/liquid ratio	0.0063	1	0.0063	18.77	0.0010 **	
AB	0.0095	1	0.0095	28.11	0.0002 **	
AC	0.0186	1	0.0186	55.10	<0.0001 **	
AD	0.0025	1	0.0025	7.39	0.0186 *	
BC	0.0030	1	0.0030	8.95	0.0113 *	
BD	0.0009	1	0.0009	2.66	0.1287	
CD	0.0146	1	0.0146	43.30	<0.0001 **	
A^2^	0.0329	1	0.0329	97.19	<0.0001 **	
B^2^	0.0776	1	0.0776	229.49	<0.0001 **	
C^2^	0.0161	1	0.0161	47.49	<0.0001 **	
D^2^	0.0533	1	0.0533	157.72	<0.0001 **	
Residual	0.0041	12	0.0003			
Lack of Fit	0.0037	10	0.0004	1.86	0.4005	not significant
Pure Error	0.0004	2	0.0002			
Cor Total	0.1657	26				
R^2^ = 0.9577			Adjusted R^2^ = 0.9469			

Note: **. The effect is highly significant, *p* < 0.01; *. The effect is significant, *p* < 0.05.

**Table 3:** Chemical constituents of volatile oil from *W. villosa* leaves.

No.	RT	Name	Structural Formula	CAS	KI	KI*	Relative Amount (%)	
							HD	Cellulase-HD
Olefins								
1	18.99	Norbornane	C10H16	497-32-5	980	940	/	0.648 ± 0.116%
2	19.24	Camphene	C10H16	79-92-5	1005	1043	1.235 ± 0.274	2.897 ± 0.114%
3	22.12	β-Phellandrene	C10H16	555-10-2	1070	1183	41.881 ± 6.262	10.634 ± 1.381%
5	23.61	β-Myrcene	C10H16	123-35-3	1114	1137	8.656 ± 2.591	/
7	23.76	β-Pinene	C10H16	18172-67-3	1125	1118	4.743 ± 1.395	/
4	24.21	α-Phellandrene	C10H16	99-83-2	1149	1164	/	0.967 ± 0.345%
6	24.71	α-Terpinene	C10H16	99-86-5	1172	1178	2.691 ± 0.780	/
8	25.73	Limonene	C10H16	5989-27-5	1206	/	8.444 ± 1.907	13.352 ± 0.264%
9	27.06	α-Terpinene	C10H16	2867-05-2	1225	1210	0.519 ± 0.082	/
10	27.24	trans-β-Ocimene	C10H16	3779-61-1	1228	1247	/	1.073 ± 0.210%
11	27.95	γ-Terpinene	C10H16	99-85-4	1237	1243	6.601 ± 1.324	14.981 ± 0.274%
12	29.84	4-Carene	C10H16	29050-33-7	1264	1149	1.541 ± 0.220	5.572 ± 0.135%
13	30.83	trans-Isolimonene	C10H16	6876-12-6	1275	/	0.044 ± 0.037	/
14	34.01	(4E,6Z)-allo-Ocimene	C10H16	7216-56-0	1325	1370	0.506 ± 0.117	0.829 ± 0.098%
15	37.24	Neo-allo-ocimene	C12H24	74630-41-4	1374	1392	/	/
16	37.38	p-Mentha-1,5,8-triene	C10H14	21195-59-5	1376	1375	0.052 ± 0.012	0.157 ± 0.005%
17	37.79	1,3,8-p-Menthatriene	C10H14	18368-95-1	1383	/	0.039 ± 0.006	0.165 ± 0.008%
18	39.65	γ-Elemene	C15H24	29873-99-2	1425	1434	0.215 ± 0.079	0.335 ± 0.097%
19	44.25	γ-Bisabolene	C15H24	242794-76-9	1561	/	0.811 ± 0.215	0.335 ± 0.097%
20	46.34	Humulene	C15H24	6753-98-6	1638	1665	/	0.411 ± 0.108%
21	47.31	Aristolochene	C15H24	26620-71-3	1707	1669	0.245 ± 0.089	0.383 ± 0.072%
22	48.21	Bicyclogermacrene	C15H24	24703-35-3	1756	1752	0.150 ± 0.063	0.220 ± 0.042%
23	48.53	epi-Bicyclosesquiphellandrene	C15H24	54274-73-6	1775	1760	/	0.213 ± 0.059%
alcohol								
24	31.26	cis-2-Penten-1-ol	C5H10O	1576-95-0	1284	1296	0.134 ± 0.068	0.293 ± 0.009%
25	32.88	1-Hexanol	C6H14O	111-27-3	1307	1325	/	0.134 ± 0.016%
26	34.51	Leaf alcohol	C6H12O	928-96-1	1332	1351	0.188 ± 0.034	0.428 ± 0.017%
27	42.08	trans-Sabinene hydrate	C10H18O	17699-16-0	1505	1483	0.093 ± 0.013	0.088 ± 0.036%
28	42.29	trans-Pinene hydrate	C10H18O	4948-29-2	1511	1432	/	0.054 ± 0.008%
29	43.28	Fenchol	C10H18O	1632-73-1	1536	1543	0.394 ± 0.105	1.843 ± 0.118%
30	44.02	4-Terpineol	C10H18O	562-74-3	1555	1552	4.974 ± 0.656	10.145 ± 0.971%
31	44.65	cis-p-Menth-2-en-1-ol	C10H18O	29803-82-5	1570	1563	0.130 ± 0.017	0.288 ± 0.016%
32	45.59	cis-Verbenol	C10H16O	18881-04-4	1593	1645	0.286 ± 0.407	/
33	46.23	trans-Verbenol	C10H16O	1820-09-3	1630	1648	0.225 ± 0.046	/
34	46.63	α-Terpineol	C10H18O	10482-56-1	1659	1690	2.751 ± 0.603	8.085 ± 0.940%
35	46.94	Borneol	C10H18O	507-70-0	1682	1698	0.675 ± 0.168	2.333 ± 0.154%
36	47.35	α-Phellandren-8-ol	C10H16O	1686-20-0	1709	1714	0.264 ± 0.067	0.486 ± 0.066%
37	47.55	cis-Carveol	C10H16O	1000374-16-8	1720	1774	0.035 ± 0.008	0.068 ± 0.010%
38	49.56	Myrtenol	C10H16O	19894-97-4	1815	1807	1.855 ± 0.422	3.073 ± 0.236%
39	50.64	4-Carenol	C10H16O	6617-35-2	1843	1816	/	0.486 ± 0.066%
40	50.77	p-Cymen-8-ol	C10H14O	1197-01-9	1847	1852	0.173 ± 0.041	0.392 ± 0.039%
ketone								
41	31.95	2,2,6-Trimethylcyclohexanone	C9H16O	2408-37-9	1293	1282	/	0.070 ± 0.006%
42	32.26	Methyl heptenone	C8H14O	110-93-0	1297	1317	0.046 ± 0.019	0.126 ± 0.024%
43	36.11	Fenchone	C10H16O	7787-20-4	1357	1383	/	0.114 ± 0.013%
44	38.18	Thujone	C10H16O	471-15-8	1389	/	/	0.079 ± 0.020%
45	41.43	1,4-Dimethyl-3-cyclohexenyl methyl ketone	C10H16O	43219-68-7	1485	1491	0.156 ± 0.043	0.484 ± 0.031%
46	41.95	2-Methyl-6-methyleneocta-1,7-dien-3-one	C10H14O	41702-60-7	1502	1345	0.177 ± 0.017	0.257 ± 0.042%
47	42.66	Isopinocamphone	C10H16O	15358-88-0	1520	1555	0.929 ± 0.125	1.417 ± 0.182%
48	43.37	Pinocarvone	C10H14O	30460-92-5	1549	1566	1.030 ± 0.139	1.580 ± 0.167%
49	45.43	Umbenone	C10H14O	24545-81-1	1590	1614	0.071 ± 0.015	0.068 ± 0.031%
50	51.23	α-Ionone	C13H20O	127-41-3	1859	1863	0.047 ± 0.018	0.112 ± 0.023%
aldehydes								
51	35.13	Nonanal	C9H18O	124-19-6	1342	1348	/	0.052 ± 0.013%
52	41.49	Benzaldehyde	C7H6O	100-52-7	1487	1480	0.196 ± 0.055	0.752 ± 0.645%
53	44.86	β-Cyclocitral	C10H16O	432-25-7	1576	1586	0.049 ± 0.006	0.094 ± 0.007%
54	45.01	α-Thujenal	C10H14O	57129-54-1	1579	/	/	0.084 ± 0.022%
55	45.23	Myrtenal	C10H14O	564-94-3	1585	1597	1.935 ± 0.316	2.740 ± 0.292%
56	47.65	Neral	C10H16O	106-26-3	1726	1733	0.137 ± 0.024	0.227 ± 0.032%
esters								
57	43.82	Isopinocampheol, acetate	C12H20O2	1000462-98-1	1549	/	0.570 ± 0.096	0.842 ± 0.141%
58	45.70	β-Sabinyl acetate	C12H18O2	3536-54-7	1597	1615	/	1.173 ± 0.106%
59	46.75	Methyl myrtenate	C11H16O2	30649-97-9	1668	1670	1.966 ± 0.403	2.727 ± 0.169%
60	49.43	Methyl acetylsalicylate	C10H10O4	580-02-9	1812	1822	0.200 ± 0.048	/
61	49.67	cis-Chrysanthenyl formate	C10H16O	1000151-75-4	1818	/	/	0.541 ± 0.025%
other								
62	29.18	o-Cymene	C10H14	527-84-4	1254	1276	1.653 ± 0.378	4.038 ± 0.090%
63	37.24	1-Methyl-1-ethylcyclopentane	C8H16	16747-50-5	1374	/	0.027 ± 0.008	/
64	37.62	β,β-Dimethylstyrene	C10H12	768-49-0	1380	/	0.137 ± 0.025	/
65	40.49	Dihydroedulan II	C13H22O	41678-32-4	1453	1492	0.124 ± 0.019	0.207 ± 0.031%

Note: RT: retention time; KI: calculated retention index; KI*: polar column retention index (retrieved from PubMed database).

**Table 4:** The ability of volatile components from *W. villosa* leaves to scavenge DPPH free radicals and ABTS free radicals.

Classification	Name	DPPH	ABTS
Olefins	Norbornane	33.39 ± 0.61%	6.46 ± 0.69%
	Camphene	34.47 ± 0.89%	11.24 ± 0.01%
	β-Phellandrene	30.71 ± 1.59%	22.45 ± 0.33%
	α-Phellandrene	43.47 ± 1.84%	3.29 ± 0.64%
	Limonene	37.04 ± 0.13%	25.93 ± 0.03%
	trans-β-Ocimene	39.46 ± 7.21%	12.45 ± 1.74%
	γ-Terpinene	40.35 ± 2.05%	36.23 ± 0.15%
	4-Carene	45.02 ± 3.35%	4.68 ± 1.58%
	(4E,6Z)-allo-Ocimene	43.09 ± 2.99%	20.12 ± 1.08%
	p-Mentha-1,5,8-triene	54.42 ± 0.34%	37.99 ± 3.63%
	1,3,8-p-Menthatriene	53.91 ± 1.69%	23.65 ± 2.82%
	γ-Elemene	78.91 ± 2.01%	57.67 ± 1.14%
	γ-Bisabolene	15.91 ± 1.74%	57.19 ± 0.45%
	Humulene	54.05 ± 0.86%	36.67 ± 4.28%
	Aristolochene	58.36 ± 0.61%	29.18 ± 1.25%
	Bicyclogermacren	55.72 ± 3.10%	/
	epi-Bicyclosesquiphellandrene	71.51 ± 3.42%	/
Alcohol	Fenchol	25.73 ± 2.69%	64.80 ± 1.97%
	4-Terpinenol	18.38 ± 1.32%	62.77 ± 5.54%
	cis-p-Menth-2-en-1-ol	24.52 ± 2.26%	/
	α-Terpineol	18.54 ± 0.30%	66.95 ± 1.11%
	Borneol	20.89 ± 1.81%	65.04 ± 2.61%
	α-Phellandren-8-ol	32.44 ± 3.15%	55.96 ± 2.41%
	Myrtenol	15.65 ± 0.33%	39.56 ± 0.99%
Ketone	Methyl heptenone	30.49 ± 3.66%	/
	1,4-Dimethyl-3-cyclohexenyl methyl ketone	43.55 ± 5.67%	66.55 ± 1.09%
	Isopinocamphone	30.34 ± 10.08%	66.58 ± 2.57%
	Pinocarvone	30.14 ± 2.82%	64.85 ± 1.75%
Aldhydes	β-Cyclocitral	38.88 ± 2.69%	/
	Myrtenal	27.54 ± 1.70%	65.57 ± 4.43%
Efters	Isopinocampheol, acetate	40.85 ± 5.05%	62.94 ± 2.79%
	Methyl myrtenate	35.15 ± 1.21%	65.03 ± 4.20%
Other	o-Cymene	55.03 ± 3.02%	20.25 ± 1.00%
	Dihydroedulan II	44.90 ± 0.37%	54.55 ± 2.66%

**Table 5:** Response surface test factors and levels (cellulase-HD).

Factor	Level		
	−1	0	1
A—Enzyme dosage (%)	1.5	2.0	2.5
B—Hydrolysis temperature (°C)	45	50	55
C—Hydrolysis time (min)	60	90	120
D—Material/liquid ratio (mL/g)	8:1	10:1	12:1

## Data Availability

Data are contained within the article.
